# Effects of Prebiotics and Probiotics on Honey Bees (*Apis mellifera*) Infected with the Microsporidian Parasite *Nosema ceranae*

**DOI:** 10.3390/microorganisms9030481

**Published:** 2021-02-25

**Authors:** Daniel Borges, Ernesto Guzman-Novoa, Paul H. Goodwin

**Affiliations:** 1 Ontario Beekeepers’ Association Technology Transfer Program, 5420 Hwy 6 N, Suite 185, Orchard Park Office, Guelph, ON N1H 6J2, Canada; dan.borges@ontariobee.com; 2 School of Environmental Sciences, University of Guelph, 50 Stone Road East, Guelph, ON N1G 2W1, Canada; pgoodwin@uoguelph.ca

**Keywords:** honey bees, prebiotics, probiotics, microbe supplements, gut microbiota, microsporidia, *Nosema ceranae*, lifespan

## Abstract

*Nosema ceranae* is a microsporidian fungus that parasitizes the midgut epithelial cells of honey bees, *Apis mellifera*. Due to the role that midgut microorganisms play in bee health and immunity, food supplementation with prebiotics and probiotics may assist in the control of *N. ceranae*. The dietary fiber prebiotics acacia gum, inulin, and fructooligosaccharides, as well as the commercial probiotics Vetafarm Probotic, Protexin Concentrate single-strain (*Enterococcus faecium*), and Protexin Concentrate multi-strain (*Lactobacillus acidophilus*, *L. plantarum*, *L. rhamnosus*, *L. delbrueckii*, *Bifidobacterium bifidum*, *Streptococcus salivarius*, and *E. faecium*) were tested for their effect on *N. ceranae* spore loads and honey bee survivorship. Bees kept in cages were inoculated with *N. ceranae* spores and single-dose treatments were administered in sugar syrup. Acacia gum caused the greatest reduction in *N. ceranae* spore numbers (67%) but also significantly increased bee mortality (62.2%). However, Protexin Concentrate single-strain gave similarly reduced spore numbers (59%) without affecting the mortality. In a second experiment, multiple doses of the probiotics revealed significantly reduced spore numbers with 2.50 mg/mL Vetafarm Probotic, and 0.25, 1.25, and 2.50 mg/mL Protexin Concentrate single-strain. Mortality was also significantly reduced with 1.25 mg/mL Protexin Concentrate single-strain. *N. ceranae*-inoculated bees fed 3.75 mg/mL Vetafarm Probotic had higher survival than *N. ceranae*-inoculated bees, which was similar to that of non-inoculated bees, while *N. ceranae*-inoculated bees fed 2.50 mg/mL Protexin Concentrate single-strain, had significantly higher survival than both *N. ceranae*-inoculated and non-inoculated bees. Protexin Concentrate single-strain is promising as it can reduce *N. ceranae* proliferation and increase bee survivorship of infected bees, even compared to healthy, non-infected bees.

## 1. Introduction

The fungus, *Nosema ceranae*, is an obligate intracellular parasite that apparently originated in the Asian honey bee *Apis cerana* and spread to the western honey bee, *Apis mellifera* [1]. Infection with this parasite occurs in the alimentary tract, and can have a number of detrimental effects on *A. mellifera*, including degeneration of the hypopharyngeal glands [2], degeneration of the midgut epithelium, reduced nutrient absorption and increased energetic stress [3,4], suppression of apoptosis [5], immunosuppression [6,7], early onset of foraging behavior [8], decreased homing and orientation [9,10], and decreased lifespan and food stores in colonies [11,12]. *N. ceranae* infections have been associated with honey bee colonies losses in North America [13,14,15] and Europe [16,17,18].

The important role that gut microorganisms play in digestive health as well as overall honey bee health and immunity may provide a means to help manage *N. ceranae* infections. The honey bee gut microbiota are comprised of a large variety of bacteria, including numerous lactic acid bacteria (LAB) within the genera *Lactobacillus* as well as bacteria in the genus *Bifidobacterium* [19,20,21,22]. The gut microbiota protects honey bees from pathogen infection by lowering the pH, competing with pathogens for nutrients and space, and producing organic acids, antimicrobial peptides (AMPs), and bacteriocins [20,21,22,23]. Nourishing and enhancing this community of microorganisms through supplementation with prebiotics and probiotics may help reduce *N. ceranae* infections.

Prebiotics are non-digestible carbohydrates and food ingredients that have been shown to increase the growth and metabolic activity of the microorganisms found in the alimentary tract, including LAB [24,25]. There have been a few studies examining prebiotic dietary fiber supplementation in bees with beneficial effects associated, such as feeding yeast-derived 1,3–1,6 β-glucan that significantly decreased deformed wing virus infections and increased honey bee survival [26], which was not surprising since glucans are immunomodulators improve innate immune responses in other organisms [27]. Also, feeding shellfish-derived chitosan increased honey bee resistance to *Nosema apis* [28]. By comparison, they have been extensively studied in other organisms. Inulin, which is found in high amounts in chicory, has been shown to increase the populations of LAB and decrease the populations of pathogenic bacterial and yeast species, as well as to prevent oxidative damage and reduce inflammation in mice intestines [29,30]. Fructooligosaccharides are also found in chicory, but are often extracted from blue agave [25]. Fructooligosaccharides can increase populations of LAB in rat and mice intestines, while decreasing populations of pathogenic bacteria [25,29,31]. In addition, fructooligosaccharides can increase the level and production of organic acids in the intestine [31]. The soluble fiber acacia gum (or gum arabic) from the trees, *Senegalia senegal* and *Vachellia seyal*, is a potent anti-inflammatory and antioxidant-promoting compound that can reduce plasma toxin levels in rats [31,32].

Probiotics are living organisms that are ingested with the goal of altering the gut microbiota [33]. By increasing beneficial microbes and reducing pathogenic species, probiotics can help prevent or treat microfauna dysbiosis resulting from disease or antibiotics [33]. There is some evidence that probiotics may help control honey bee parasites. Feeding honey bee colonies *Bacillus subtilis*, an endogenous gut bacterium, reduced *Nosema* spp. spore counts compared to the control throughout an eight-month study, although reduced spore counts were only significant during two of those months [34]. Feeding caged bees the honey bee gut bacterium *L. kunkeei* reduced *N. ceranae* spore loads compared to control untreated bees [35] as did feeding another honey bee gut bacterium, *Parasaccharibacter apium*, to bees in hives [36], while isolated gut bacterial strains and the commercial probiotics, Bactocell^®^ and Levucell SB^®^, increased survival of *Nosema*-infected bees above that of uninfected control bees [37]. In contrast, feeding infected caged bees a mixture of *L. casei*, *L. plantarum*, *Saccharomyces cerevisiae*, and *Rhodopseudomonas palustris* caused a significant increase in *Nosema* spore counts compared to the infected control bees [38], and feeding the honey bee gut bacterium, *Snodgrassella alvi* to bees in hives, increased their susceptibility to the protozoan parasite, *Lotmaria passim*, that was believed to be due to it causing dysbiosis of the gut microbiota [39]. Thus, one cannot assume that all potential probiotics will provide benefits to honey bees.

Formulated commercial probiotics have already been shown to be effective in other animals [40,41]. For example, Vetafarm Probotic was developed for caged birds and poultry, and contains the bacterial species, *L. acidophilus*, *L. plantarum*, *L. rhamnosus*, *L. delbrueckii* subspecies *bulgaricus*, *Bifidobacterium bifidum*, *Streptococcus salivarius* subspecies *thermophilus*, and *Enterococcus faecium*, all at a concentration of 1.80 × 10^8^ CFU/g. Two other probiotic formulations, Protexin Concentrate single-strain and Protexin Concentrate multi-strain, were developed for poultry, pigs, sheep, goats, and cattle. Protexin Concentrate single-strain contains 2.00 × 10^9^ CFU/g of only *E. faecium*, while Protexin Concentrate multi-strain contains the same seven species of bacteria as Vetafarm Probotic but at a higher concentration of 2.00 × 10^9^ CFU/g. The replacement of endogenous bacteria with supplemented bacteria may explain some of the beneficial effects of commercial probiotics. 

In this study, three prebiotics, acacia gum, inulin, and fructooligosaccharides, and three commercial veterinary probiotics, Vetafarm Probotic, Protexin Concentrate single-strain, and Protexin Concentrate multi-strain, were examined for their effects on reducing *N. ceranae* spore counts and increasing longevity of infected honey bees.

## 2. Materials and Methods

### 2.1. Ethical Statement

This study was conducted under the supervision of researchers of the Honey Bee Research Centre, University of Guelph, Guelph ON, Canada. Beekeeping practices were in compliance with the Ontario Ministry of Agriculture, Food and Rural Affairs (OMAFRA) bio-safety regulations. No permits were required to conduct the study.

### 2.2. Nosema ceranae Spore Extraction

Forager honey bees were collected with a bee vacuum [42] from hives kept at the Honey Bee Research Centre, University of Guelph. *N. ceranae* spores were detected and quantified by microscopy [43] to identify colonies that were highly infected. Bees collected from the most infected colonies were pooled and stored at −20 °C, and the spores were extracted and used for experiments within 24 h. 

To extract *N. ceranae* spores, bee abdomens were macerated in 25 mL of dH_2_O with a mortar and pestle. The macerate was filtered through a honey filter of 177 μm (Better Bee Supplies, Cambridge, ON, Canada). Then, the macerate was centrifuged for 8 min at 800× *g*. The supernatant was discarded, and the remaining macerate was combined in a 2 mL tube and vortexed for 10 s. DNA was extracted from the spores and the presence of only *N. ceranae* was confirmed by PCR analysis as per Hamiduzzaman et al. [44].

### 2.3. Inoculation of Honey Bees with Nosema ceranae Spores

Honey bees were inoculated with *N. ceranae* spores [45]. Briefly, frames with capped brood from colonies without *N. ceranae* infection were maintained in an incubator overnight, and newly emerged bees were collected the following day. Bees were starved for 2 h and diagnosis of *N. ceranae* was performed using 10 bees to ensure that the newly emerged bees had no detectable *N. ceranae* spores. Extracted spores were quantified with a hemocytometer and diluted to 10,000 spores/µL in 50% sugar syrup. Then, bees were individually fed 5 µL of the sugar syrup containing extracted spores using a micropipette (Eppendorf, Mississauga, ON, Canada). Each bee was inoculated with approximately 50,000 spores to infect >98% of the bees [46]. Bees that did not consume the entire 5 µL of inoculum were discarded. After feeding, batches of 40 bees were placed in wooden hoarding cages (13.0 × 9.5 × 15 cm) and maintained in an incubator at 30 °C and 65% relative humidity. Bees of the negative control were individually fed 5 µL of sugar syrup without spores.

### 2.4. Prebiotic Compounds and Probiotic Formulas

The prebiotics inulin, fructooligosaccharides, and gum arabic were obtained from Sigma-Aldrich (Oakville, ON, Canada). The Vetafarm Probotic was obtained from Vetafarm (Wagga Wagga, NSW, Australia). The probiotics Protexin Concentrate single-strain and Protexin Concentrate multi-strain were obtained from Probiotics International Limited (Lopen, Somerset, UK). Because the prebiotic compounds had not previously been tested on bees, doses were determined by looking at studies on other organisms, and adjusting the dose based on an average body weight of 100 mg per bee or average feed consumption over 16 days. For the probiotics, the feeding guide on the packaging (Vetafarm Probotic) or the manufacturer’s website (Protexin Concentrate single-strain and Protexin Concentrate multi-strain) was used. The appropriate dose for a bee was calculated using an average body weight of 100 mg per bee. The doses used are shown in Table 1.

### 2.5. Prebiotic and Probiotic Screening

Bees in each cage were administered one prebiotic compound or probiotic formula mixed in 50% sucrose syrup, which was administered to the bees in 15 mL drip feeders. Therefore, three cages received the prebiotics and three cages received the probiotics to be tested. Non-inoculated, negative control and inoculated, positive control bees were both provided with feeders containing only 50% sucrose syrup. Feeders containing water were also provided for all cages. Feeders were changed every four days and weighed before and after changing using a balance (Model S-403, Denver Instrument, Bohemia, New York, NY, USA) to determine the feed and water consumption. The average number of bees alive between each feeder change and the amount of syrup or water consumed was used to estimate the total intake per bee. Dead bees were removed daily and counted. At 16 days post-inoculation (dpi), remaining bees were stored at −20 °C, and spore counts were done using a hemocytometer. Bee mortality was calculated at 16 dpi excluding bees that died within 2 dpi, as this is typically due to handling and inoculation stress and not *N. ceranae* infection. The experiment was replicated three times.

### 2.6. Comparing Doses of Selected Probiotics

Using the procedure above, two doses of Protexin Concentrate multi-strain (0.25 and 1.25 mg/mL), three doses of Vetafarm Probotic (0.50, 2.50, and 3.75 mg/mL) and three doses of Protexin Concentrate single-strain (0.25, 1.25, and 2.50 mg/mL) were compared. The experiment was replicated three times.

### 2.7. Long-Term Bee Mortality with Selected Probiotics

To assess long-term bee mortality with Vetafarm Probotic and Protexin Concentrate single-strain treatments, the above procedures were employed, except that the experiment continued until every bee had died. Spore counts were performed on dead bees to ensure that the inoculation was successful and that bees were infected (data not shown). Survivorship curves were created for each treatment using the mortality data for individual bees within a particular treatment cage. Bees that died within 2 dpi were excluded from the analysis.

### 2.8. Statistical Analyses 

Using Kolmogorov–Smirnov tests of normality, data were found to be normally distributed except for spore counts and percent mortality, which were Log10 transformed and arcsine-square root transformed before analyses, respectively. Analyses of variance (ANOVA) were used to compare the treatments and when significant effects were found, means were separated by Fisher’s LSD tests (*p* = 0.05). Results were expressed as means ± S.E. Best-fit line models were used to examine the relationship between doses of the three probiotics and spore numbers, mortality, feed intake, and water intake. As responses to doses were pooled from the primary screening and dose response experiments, the positive control values were pooled for the analyses. ANOVA, Fisher’s LSD, and line of best-fit analyses were done using SAS^®^ Studio version 3.8 (SAS Institute, Cary, NC, USA). Regression analyses and best-fit graphs were done with Excel^®^ for Office 365 MSO version 2002 (Microsoft, Redmond, Washington, DC, USA). Kaplan–Meier survival curves for the bees in each treatment were created and compared using a log-rank/Mantel-Cox post-hoc test to determine the significant differences using the SPSS version 22 (IBM SPSS Statistics, Armonk, New York, NY, USA). All tests used a Type I error of 0.05 to determine the significance. 

## 3. Results

### 3.1. Prebiotic and Probiotic Screening

*N. ceranae* spores were not detected in the negative control, but they were found in the inoculated bees in the rest of the treatments, indicating the infection resulted from inoculation. Compared to the positive control, acacia gum, Protexin Concentrate single-strain, Protexin Concentrate multi-strain, and fructooligosaccharides treatments resulted in significantly lower spore numbers (F_7,81_ = 971.08, *p* < 0.0001) with 67%, 59%, 34%, and 31% reductions, respectively (Table 2). However, bee mortality was significantly higher with acacia gum compared to the controls, unlike all the other treatments (F_7,28_ = 6.62, *p* < 0.001; Table 3). While feed intake was notably higher for acacia gum and Vetafarm Probotic, no significant differences were found between treatments (F_7,28_ = 1.85, *p* = 0.130; Table 4). Water intake was approximately doubled with acacia gum and Protexin Concentrate multi-strain compared to the control, but no significant differences were observed (F_7,27_ = 1.94, *p* = 0.115; Table 4). 

Based on the results, acacia gum was not further examined as it caused high mortality, despite reducing *N. ceranae* spore numbers, while Protexin Concentrate single-strain and Protexin Concentrate multi-strain were considered promising as they gave the next highest reductions in spore numbers with similar bee mortality as the negative control. Even though it did not result in significant differences in spore numbers from the positive control, Vetafarm Probotic was further studied to determine if its effectiveness in decreasing bee mortality could be increased.

### 3.2. Response to Different Doses of Selected Probiotics

**Figure 1:**
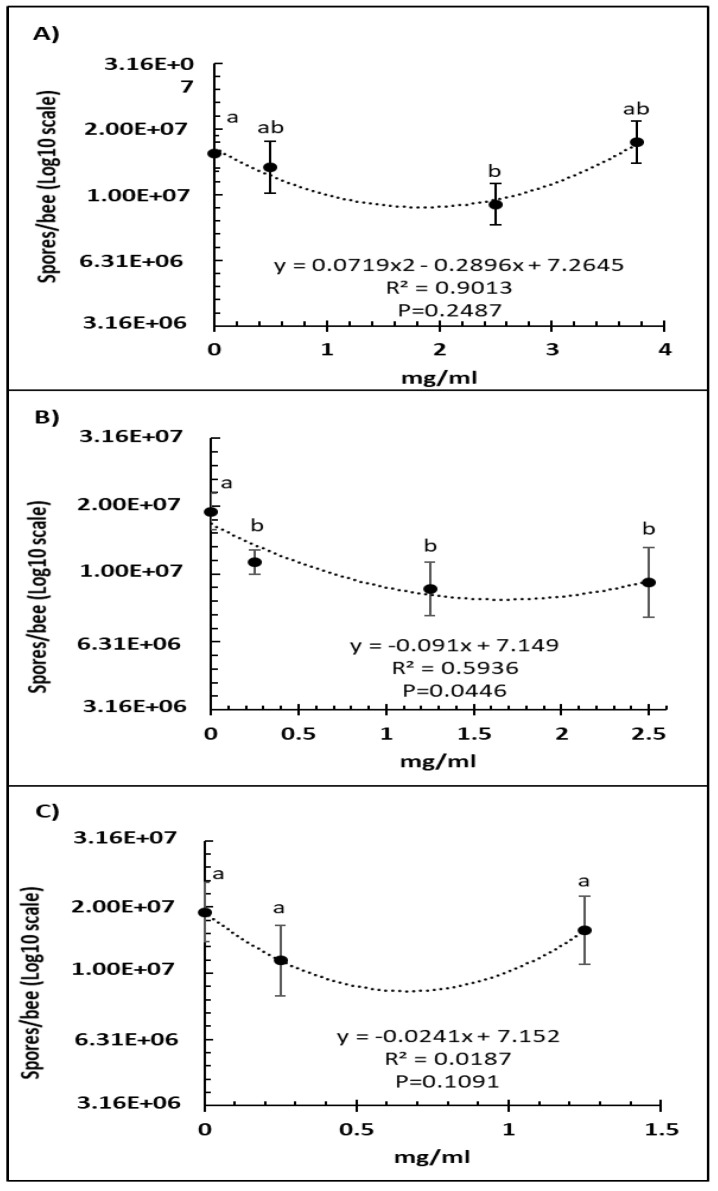
Relationship between *N. ceranae* spore count (Log10) and concentration (mg/mL) of the probiotics Vetafarm Probotic (**A**), Protexin Concentrate single-strain (**B**), and Protexin Concentrate multi-strain (**C**), fed to *N. ceranae*-infected honey bees. Treatments followed by the same letter are not significantly different.

A comparison of several doses of Vetafarm Probotic showed that only 2.50 mg/mL resulted in significantly lower number of *N. ceranae* spores than the positive control not treated with the probiotic (F_3,44_ = 4.99, *p* < 0.01), and a higher dose was less effective (Figure 1A). For Protexin Concentrate single-strain, all the doses resulted in significantly lower spore numbers than the positive control with no significant differences between each dose (F_3,42_ = 3.58, *p* = 0.022; Figure 1B). The line of best fit for spore count versus Protexin Concentrate single-strain dose was significant. A comparison of Protexin Concentrate multi-strain doses showed that none resulted in significantly lower spore numbers than the non-treated positive control (F_2,34_ = 2.182, *p* = 0.129; Figure 1C). 

**Figure 2:**
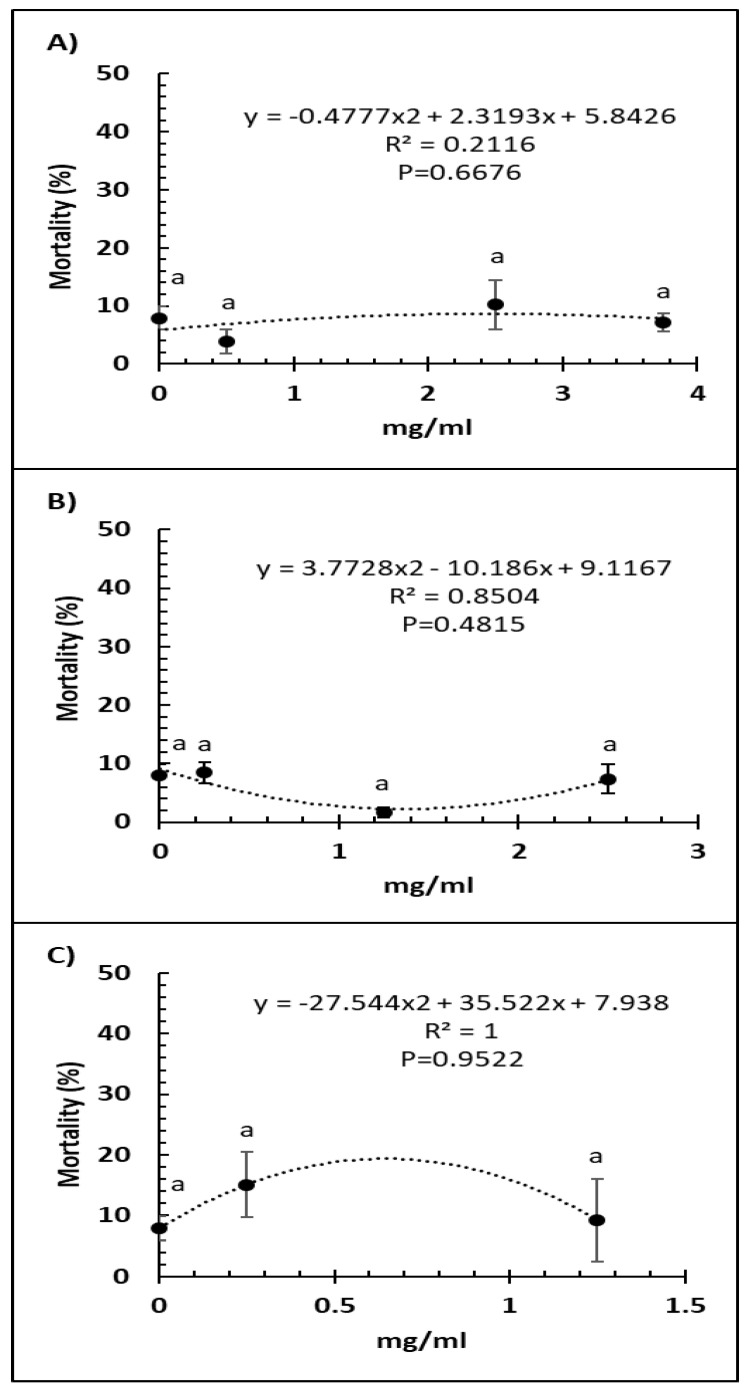
Relationship between percent bee mortality and concentration (mg/mL) of the probiotics Vetafarm Probotic (**A**), Protexin Concentrate single-strain (**B**), and Protexin Concentrate multi-strain (**C**), fed to *N. ceranae*-infected honey bees. Treatments followed by the same letter are not significantly different.

Bee mortality was not significantly altered by any of the doses of Vetafarm Probotic (F_3,14_ = 0.884, *p* = 0.479; Figure 2A). Bee mortality was not significantly lower with any of the doses of Protexin Concentrate single-strain compared to the positive control (F_3,14_ = 1.950, *p* = 0.180; Figure 2B). No dose of Protexin Concentrate multi-strain significantly changed bee mortality (F_2,11_ = 0.835, *p* = 0.465; Figure 2C).

**Figure 3:**
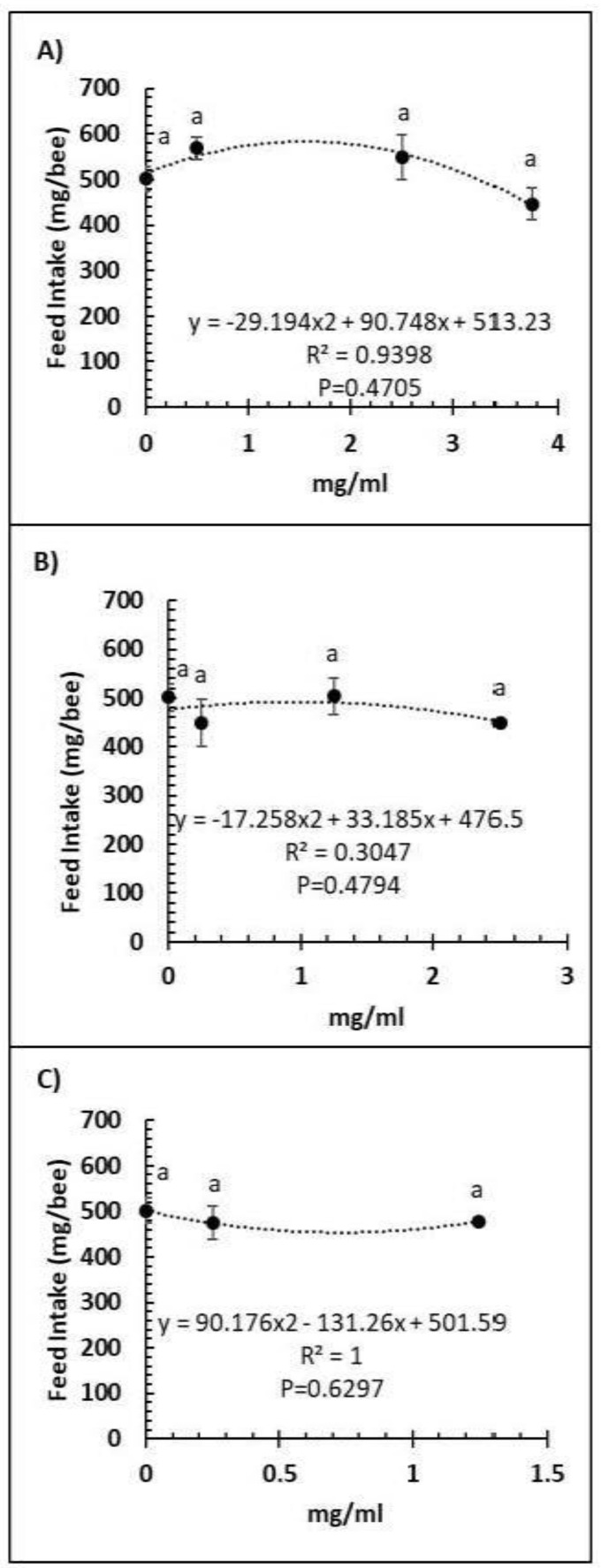
Relationship between feed intake and concentration (mg/mL) of the probiotics Vetafarm Probotic (**A**), Protexin Concentrate single-strain (**B**), and Protexin Concentrate multi-strain (**C**), fed to *N. ceranae*-infected honey bees. Treatments followed by the same letter are not significantly different.

**Figure 4:**
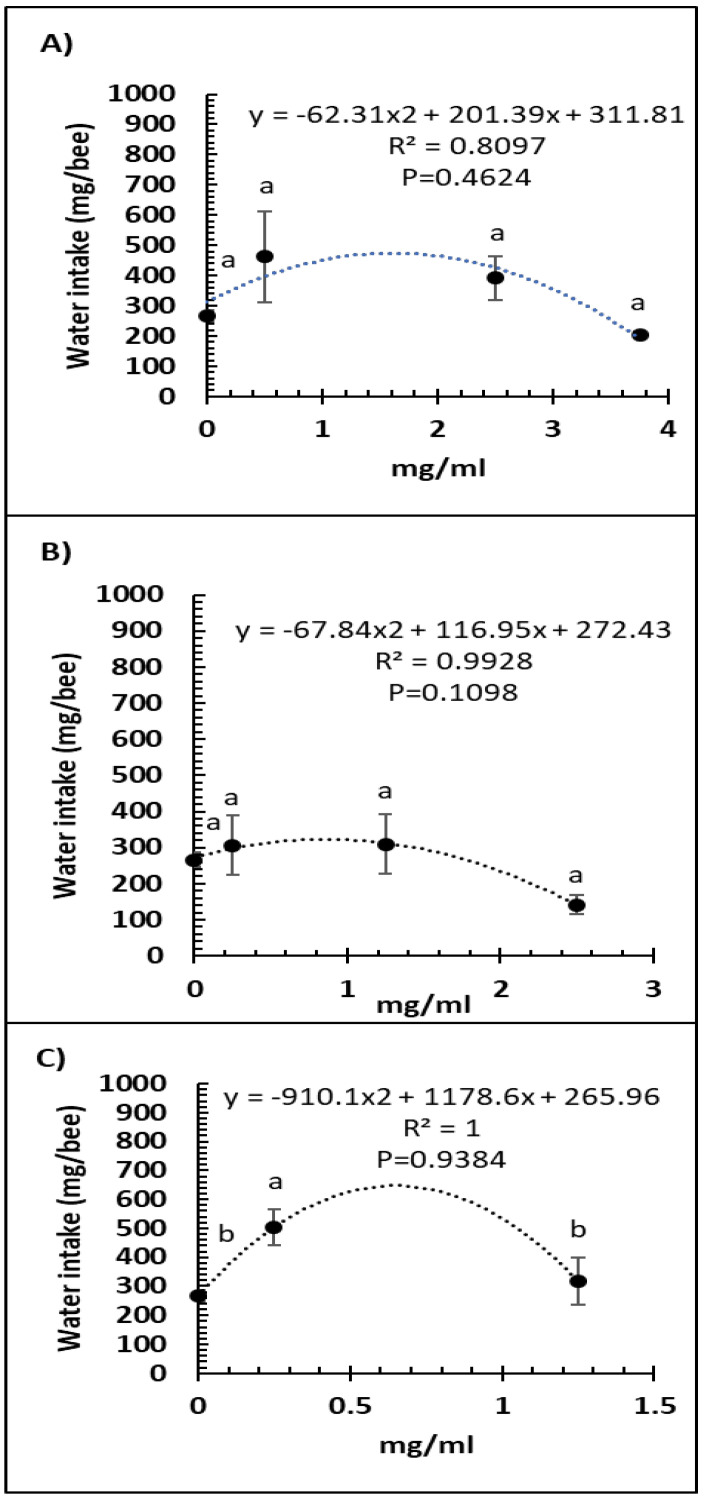
Relationship between water intake and concentration (mg/mL) of the probiotics Vetafarm Probotic (**A**), Protexin Concentrate single-strain (**B**), and Protexin Concentrate multi-strain (**C**), fed to *N. ceranae*-infected honey bees. Treatments followed by the same letter are not significantly different.

A comparison of feed intake and dose of Vetafarm Probotic, Protexin Concentrate single-strain, or Protexin Concentrate multi-strain showed that none gave significantly altered feed intake (F_3,14_ = 1.110, F_3,14_ = 0.779, F_2,11_ = 0.275, *p* >0.05, respectively; Figure 3A–C). Water intake was not significantly affected by any of the tested doses of Vetafarm Probotic or Protexin Concentrate single-strain (F_3,12_ = 2.199, F_3,12_ = 1.869, *p* >0.05, respectively; Figure 4A,B). However, water intake was significantly higher with 0.25 mg/mL Protexin Concentrate multi-strain (F_2,9_ = 5.330, *p* = 0.039; Figure 4C).

### 3.3. Survivorship of Treated Bees

**Figure 5:**
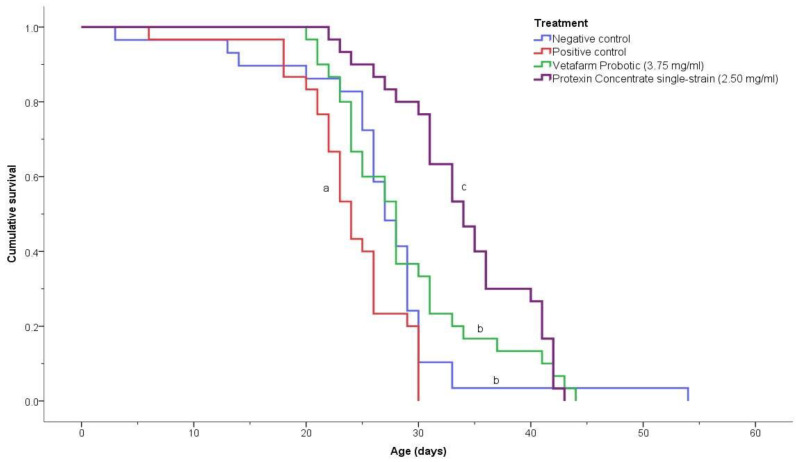
Kaplan-Meier survival curves for *N. ceranae*-infected honey bees fed the probiotics Vetafarm Probotic and Protexin Concentrate single-strain. Curves labelled with the same letter are not significantly different.

The negative control, positive control, 3.75 mg/mL Vetafarm Probotic, and 2.50 mg/mL Protexin Concentrate single-strain, showed significant differences for the survival curves (χ^2^ = 36.190, *p* < 0.00001; Figure 5). The positive control bees had a significantly lower survival rate than the bees of all other treatments. The negative control bees and the bees fed Vetafarm Probotic had higher survival curves than the positive control, and were significantly different from other treatments, although not significantly different from one another. Bees fed Protexin Concentrate single-strain had the highest survival, which was significantly higher than all the other treatments, including the negative control. Thus, *N. ceranae*-inoculated bees treated with Vetafarm Probotic had similar survival to non-inoculated bees, and *N. ceranae*-inoculated bees treated with Protexin Concentrate single-strain had even higher survival than non-inoculated bees.

## 4. Discussion

Screening a number of prebiotics and probiotics for their potential in controlling *N. ceranae* infections in honey bees showed that acacia gum, Protexin Concentrate single-strain, Protexin Concentrate multi-strain, and fructooligosaccharide treatments resulted in significant reductions of 67 to 31% in *N. ceranae* spore numbers. These results are in agreement with recent studies that have found other natural compounds such as natural plant extracts, mannan-oligosaccharide, cineol, zymosan, citral, naringenin, carvacrol, sulforaphane, chitosan, and peptidoglycan, that could reduce *N. ceranae* spore counts between 49 and 95% [12,47,48]. 

In this study, the most effective compound in controlling *N. ceranae* infections was acacia gum. Ali et al. [32] found that acacia gum had strong anti-inflammatory and antioxidant properties in rats. Feeding the animals with acacia gum in water resulted in decreased expression of pro-inflammatory cytokines and decreased reactive oxygen species (ROS) generation. Being a prebiotic dietary fiber that is utilized by gut bacteria, acacia gum can also stimulate growth and development of gut microbiota, resulting in improved digestion and absorption, decreased colonization by pathogenic bacteria, and increased populations of beneficial bacteria and the AMPs and organic acids that they produce [24]. Ballal et al. [49] found that acacia gum could reduce the growth of the apicomplexan malaria-causing parasite *Plasmodium falciparum* and reduced mortality and parasite growth of *Plasmodium berghei* when fed to mice. The authors hypothesized that this may result from increased production of organic acids by the gut bacteria, which can stimulate the immune system and increase phagocytosis of infected cells. Thus, acacia gum may have had several impacts on the bees, leading to lower *N. ceranae* spores.

Among the other two prebiotics tested in this study, inulin did not show a significant decrease in *N. ceranae* spores, and fructooligosaccharides resulted in the lowest significant decrease among the compounds tested. Inulin and fructooligosaccharides are dietary fibers that can improve intestinal health and stimulate growth of gut microbiota [29,30]. However, inulin and fructooligosaccharides have not been shown to have anti-parasite properties like acacia gum, and this possibly explains their limited effectiveness, in reducing spore numbers.

The three prebiotics tested in this study resulted in the highest bee mortality of all treatments, with acacia gum being significantly more toxic than the controls and other treatments. While this may seem contradictory to other findings on prebiotic supplementation, Younes et al. [31] found that feeding acacia gum and fructooligosaccharides to rats, led to an increased nitrogen transport into the intestine in the form of blood urea. Bacterial urease enzymes converted the urea to ammonia, which was used for microbial growth and protein synthesis. The overall effect was to increase fecal excretion of nitrogen, increase fecal bulk, and accelerate transit of feces. It is possible that feeding bees prebiotics also led to increased nitrogen uptake from bee frass because caged bees do not defecate and could not excrete the nitrogenous wastes. As a result, the accumulation of nitrogenous wastes in the intestine may have been toxic, and increased defecation stimulus without defecation may have put additional stress on the bees. Increased ammonia in the gut may have also increased gut pH and counteracted the acidification by organic acids from beneficial LAB that help improve intestinal health and function [50]. While high bee mortality rates were seen with all the prebiotics, it would be worthwhile to see if this also occurred in a field setting where bees would be able to defecate. 

In addition to prebiotics, commercial probiotics were tested. Several studies of probiotics in honey bees using probiotic bacteria isolated from honey bee guts have shown activity against *N. ceranae*. The bacteria *L. johnsonii* has antimicrobial activity against a number of bacteria and fungi and produces large amounts of lactic acid [20]. Feeding colonies with *L. johnsonii* resulted in increased brood production, adult bee population, and honey storage [20]. Feeding colonies with organic acids produced by *L. johnsonii* also resulted in increased adult bee population, higher winter survival of colonies, and bees with more developed fat bodies, as well as *N. ceranae* spore numbers approximately 50% lower than in control colonies [51]. Feeding colonies *B. subtilis* spores from an isolate from honey bee guts increased brood production, adult population, and honey storage [34]. In addition, colonies fed *B. subtilis* had significantly lower *Nosema* spp. spore numbers in two out of eight months compared to the control. Porrini et al. [52] found that the surfactin of *B. subtilis* effective against *N. ceranae* reduced spore viability by almost 50% and significantly reduced bee mortality compared to infected control bees. 

*E. faecium* is another species of bacteria isolated from the guts of many other animals. This species of bacterium is found in all three probiotic formulas tested in this study. The other bacterial species isolated from honey bee guts or hive environment that were present in both Vetafarm Probotic and Protexin Concentrate multi-strain are *L. acidophilus* and *L. plantarum* [20,53]. Like *E. faecium*, these two species of bacteria are found in the guts of a variety of other organisms as well as in many fermented foods and have been widely used as probiotics. Commercial probiotics containing *L. acidophilus*, *Bifidobacterium lacti*, or *L. casei* have increased honey bee colony health and production parameters [54], as well as decreased the populations of gut bacteria and increased the populations of beneficial LAB [55]. Feeding other commercial probiotics to bees has also increased expression and production of AMPs, such as abaecin and hymenoptaecin [56,57]. In rats, probiotics increased intestinal epithelial regeneration after chemotherapy treatment [58]. This may be particularly beneficial in *N. ceranae*-infected bees where epithelial degeneration occurs and regeneration is suppressed by the pathogen [3,4].

The benefits observed with the probiotics tested in this study may be due to a combination of their antimicrobial and intestinal health activities. As Protexin Concentrate single-strain showed higher percent reduction in spore numbers than the other two formulas and yet only contained *E. faecium*, much of this antimicrobial effect may have been due to *E. faecium*. While *E. faecium* can produce a number of antimicrobial compounds [20,59], not all antimicrobial compounds are necessarily effective against *N. ceranae*. For example, Porrini et al. [52] tested two bacteriocins isolated from *E. faecium* against *N. ceranae*, both in vitro and in vivo, and neither reduced spore viability or bee mortality in infected bees. Thus, it is possible that they have only antibacterial action, and it is other compounds produced by *E. faecium* that have activity against microsporidia and other fungi.

*Enterococcus faecium* can prevent intestinal colonization by pathogens [41]. It produces lactic acid, although at lower levels than *L. johnsonii* [20]. Other organic acids commonly produced by gut bacteria include acetic acid and butyric acid [25,47]. Organic acids produced by gut bacteria cause thickening of the peritrophic membrane in honey bees [51]. As *N. ceranae* spores must first pass through the peritrophic membrane to begin infection of midgut epithelial cells, thickening of the peritrophic membrane may help prevent infections from the start. This may be particularly important as *N. ceranae* infection causes fragmentation of the chitin-rich peritrophic membrane, possibly through the up-regulation of chitinase genes [5,60].

One way that probiotics may improve intestinal health is by shifting populations of other intestinal microbes. Feeding a probiotic mixture of *L. acidophilus, L. casei*, and *Bifidobacterium lactis* to honey bee colonies significantly reduced the number of bacteria in the gut, resulting in a higher percentage of beneficial LAB from the probiotic mixture, compared to the control [55]. This indicates that bacteria in probiotics can outcompete and replace other intestinal bacterial species as they colonize the gut. Thus, the beneficial effects of *E. faecium* in Protexin Concentrate single-strain could be indirect by shifting populations of other honey bee gut microbiota that ultimately inhibit *N. ceranae* infection. However, *N. ceranae* only infects the midgut, which showed little change in the microbiota related to the intensity of *N. ceranae* infection, except for a positive correlation with two *Gilliamella* sequence variants [61]. Possibly the effects of *E. faecium* in Protexin Concentrate single-strain as well as other probiotics against *N. ceranae* are primarily related to their impacts on the metabolism of the midgut cells or the metabolism of the midgut microbiome rather than altering the midgut microbiome structure. Further work is needed to examine the molecular and cellular effects of feeding probiotics in this study, including examining their effects in newly emerged bees allowed to eat bee bread to establish a gut microbiome before treatment, which was not done in this study.

A metabolic effect may explain why Protexin Concentrate single-strain worked better than Protexin Concentrate multi-strain. As Protexin Concentrate single-strain contains only *E. faecium*, it may have had a more targeted impact than Protexin Concentrate multi-strain and Vetafarm Probotic, each containing *E. faecium* and six additional species, each of which would be producing a different range of metabolites having different impacts. In addition, one or more of these species may have had negative effects, which may explain why the highest dose of Vetafarm Probotic was less effective than the intermediate dose, and why Protexin Concentrate multi-strain was less effective than Vetafarm Probotic, even though they both contained the same species but with higher concentrations of bacteria in Protexin Concentrate multi-strain. There was no evidence that Protexin Concentrate single-strain had any negative effects at the doses tested, indicating that negative effects may be related to other bacteria in the multi-species probiotic mixtures. Further work should examine all the bacteria in these formulations separately to identify if any are directly or indirectly harmful to honey bees. 

In conclusion, two probiotic formulas, Vetafarm Probotic and Protexin Concentrate single-strain, showed promise as alternative controls for *N. ceranae* infection in *A. mellifera*. In particular, Protexin Concentrate single-strain treatment both reduced *N. ceranae* spore numbers and increased infected honey bee survival above that of both infected control bees and non-infected control bees. The prebiotic acacia gum may also be promising if its toxicity to bees can be reduced, such as in the field, where bees are able to fly and defecate. Better understanding the modes of action of these prebiotics and probiotics will be needed to optimize their effectiveness.

## Figures and Tables

**Table 1:** Doses (mg/mL) of prebiotic compounds and probiotic formulas used for a screening experiment, and the source for each dose. Doses for prebiotics were calculated from the study doses using an average body weight of 100 mg per bee. Doses for probiotics were calculated from the manufacturer’s instructions, also using an average body weight of 100 mg per bee. All doses were prepared in 50% sucrose syrup.

Treatment	Dose	Source	Method	Species
Acacia gum	41.667	Ballal et al., 2011 [42]	Water	Mice
Inulin	20.833	Buddington et al., 2002	Feed	Mice
Fructooligosaccharides	20.833	Buddington et al., 2002	Feed	Mice
Protexin Concentrate single-strain	0.2500	Manufacturer	Feed	Chickens
Protexin Concentrate multi-strain	0.2500	Manufacturer	Feed	Chickens
Vetafarm Probotic	0.5000	Manufacturer	Feed	Chickens

**Table 2:** Mean *N. ceranae* spore number per bee ± SE of non-infected (negative control) or infected bees fed sugar syrup (positive control) or sugar syrup containing prebiotics or probiotics.

Treatment	Mean Spore Number (Spores/Bee ± SE)	Means Comparison ^1^
Negative control	0.00 × 10^0^ ± 0.00 × 10^0^	d
Positive control	1.70 × 10^7^ ± 2.06 × 10^6^	a
Acacia gum	5.58 × 10^6^ ± 1.08 × 10^6^	c
Inulin	1.34 × 10^7^ ± 3.48 × 10^6^	a,b
Protexin Concentrate single-strain	7.04 × 10^6^ ± 7.28 × 10^5^	c
Protexin Concentrate multi-strain	1.12 × 10^7^ ± 2.05 × 10^6^	b
Fructooligosaccharides	1.17 × 10^7^ ± 1.64 × 10^6^	b
Vetafarm Probotic	1.54 × 10^7^ ± 3.18 × 10^6^	a,b

^1^ Treatments followed by the same letter are not significantly different based on ANOVA and Fisher’s LSD tests on Log10 transformed data.

**Table 3:** Mean bee mortality ± SE (%) of non-infected (negative control) and *N. ceranae*-infected bees fed sugar syrup (positive control) or sugar syrup containing prebiotics or probiotics.

Treatment	Mortality (% ± SE) ^1^	Means Comparison ^2^
Negative control	6.15 ± 0.0	b
Positive control	7.94 ± 1.96	b
Acacia gum	62.2 ± 18.3	a
Inulin	16.6 ± 7.96	b
Fructooligosaccharides	15.9 ± 9.12	b
Protexin Concentrate single-strain	8.44 ± 1.87	b
Protexin Concentrate multi-strain	7.36 ± 5.38	b
Vetafarm Probotic	3.86 ± 2.12	b

^1^ Bees that died within the first 48 h were excluded from the analysis. ^2^ Treatments followed by the same letter are not significantly different based on ANOVA and Fisher’s LSD tests on arcsine square root transformed data.

**Table 4:** Mean feed and water intake ± SE (mg of syrup or water/bee in 16 days) of non-infected (negative control) and *N. ceranae*-infected bees fed sugar syrup (positive control) or sugar syrup containing prebiotics or probiotics.

Treatment	Mean Feed Intake (mg/Bee ± SE)	Mean Water Intake (mg/Bee ± SE)
Negative control	524.9 ± 22.0	245.1 ± 48.7
Positive control	501.6 ± 27.7	266.0 ± 19.2
Acacia gum	533.2 ± 83.9	509.0 ± 77.4
Inulin	444.4 ± 17.8	347.4 ± 89.6
Fructooligosaccharides	393.5 ± 40.3	438.4 ± 119.2
Protexin Concentrate single-strain	448.9 ± 24.9	306.4 ± 82.1
Protexin Concentrate multi-strain	474.5 ± 36.8	503.7 ± 60.6
Vetafarm Probotic	568.5 ± 52.5	463.3 ± 150.8

## Data Availability

The data presented in this study will be made available upon reasonable request from the corresponding author.

## References

[1] Goblirsch M. (2018). *Nosema ceranae* disease of the honey bee (*Apis mellifera*). Apidologie.

[2] Martín-Hernández R., Bartolomé C., Chejanovsky N., Le Conte Y., Dalmon A., Dussaubat C., García-Palencia P., Meana A., Pinto M.A., Soroker V. (2018). *Nosema ceranae* in *Apis mellifera*: A 12 years postdetection perspective. Environ. Microbiol..

[3] Dussaubat C., Brunet J.L., Higes M., Colbourne J.K., Lopez J., Choi J.H., Martín-Hernández R., Botías C., Cousin M., McDonnell C. (2012). Gut pathology and responses to the Microsporidium *Nosema ceranae* in the honey bee *Apis mellifera*. PLoS ONE.

[4] Martín-Hernández R., Botías C., Barrios L., Martínez-Salvador A., Meana A., Mayack C., Higes M. (2011). Comparison of the energetic stress associated with experimental *Nosema ceranae* and *Nosema apis* infection of honeybees (*Apis mellifera*). Parasitol. Res..

[5] Higes M., Juarranz A., Dias-Almeida J., Lucena S., Botías C., Meana A., García-Palencia P., Martín-Hernández R. (2013). Apoptosis in the pathogenesis of *Nosema ceranae* (Microsporidia: Nosematidae) in honey bees (*Apis mellifera*). Environ. Microbiol. Rep..

[6] Antúnez K., Martín-Hernández R., Prieto L., Meana A., Zunino P., Higes M. (2009). Immune suppression in the honey bee (*Apis mellifera*) following infection by *Nosema ceranae* (Microsporidia). Environ. Microbiol..

[7] Chaimanee V., Chantawannakul P., Chen Y., Evans J.D., Pettis J.S. (2012). Differential expression of immune genes of adult honey bee (*Apis mellifera*) after inoculated by *Nosema ceranae*. J. Insect Physiol..

[8] Goblirsch M., Huang Z.Y., Spivak M. (2013). Physiological and behavioral changes in honey bees (*Apis mellifera*) induced by *Nosema ceranae* infection. PLoS ONE.

[9] Dussaubat C., Maisonnasse A., Crauser D., Beslay D., Costagliola G., Soubeyrand S., Kretzchmar A., Le Conte Y. (2013). Flight behavior and pheromone changes associated to *Nosema ceranae* infection of honey bee workers (*Apis mellifera*) in field conditions. J. Invertebr. Pathol..

[10] Wolf S., McMahon D.P., Lim K.S., Pull C.D., Clark S.J., Paxton R.J., Osborne J.L. (2014). So near and yet so far: Harmonic radar reveals reduced homing ability of *Nosema* infected honeybees. PLoS ONE.

[11] Emsen B., De la Mora A., Lacey B., Eccles L., Kelly P.G., Medina-Flores C.A., Petukhova T., Morfin N., Guzman-Novoa E. (2020). Seasonality of *Nosema ceranae* infections and their relationship with honey bee populations, food stores, and survivorship in a North American region. Vet. Sci..

[12] Valizadeh P., Guzman-Novoa E., Goodwin P.H. (2020). Effect of immune inducers on *Nosema ceranae* multiplication and their impact on honey bee (*Apis mellifera* L.) survivorship and behaviors. Insects.

[13] Cox-Foster D.L., Conlan S., Holmes E.C., Palacios G., Evans J.D., Moran N.A., Quan P.L., Briese T., Hornig M., Geiser D.M. (2007). A Metagenomic survey of microbes in honey bee Colony Collapse Disorder. Science.

[14] Currie R.W., Pernal S.F., Guzman-Novoa E. (2010). Honey bee colony losses in Canada. J. Apic. Res..

[15] Van Engelsdorp D., Evans J.D., Saegerman C., Mullin C., Haubruge E., Nguyen B.K., Frazier M., Frazier J., Cox-Foster D., Chen Y. (2009). Colony collapse disorder: A descriptive study. PLoS ONE.

[16] Dainat B., Evans J.D., Chen Y.P., Gauthier L., Neumann P. (2012). Predictive markers of honey bee colony collapse. PLoS ONE.

[17] Higes M., Martín-Hernández R., Botías C., Garrido-Bailón E., González-Porto A.V., Barrios L., del Nozal M.J., Bernal J.L., Jiménez J.J., García-Palencia P. (2008). How natural infection by *Nosema ceranae* causes honeybee colony collapse. Environ. Microbiol..

[18] Higes M., Meana A., Bartolomé C., Botías C., Martín-Hernández R. (2013). *Nosema ceranae* (Microsporidia), a controversial 21st century honey bee pathogen. Environ. Microbiol. Rep..

[19] Anderson K.E., Johansson A., Sheehan T.H., Mott B.M., Corby-Harris V., Johnstone L., Sprissler R., Fitz W. (2013). Draft genome sequences of two *Bifidobacterium* sp. from the honey bee (*Apis mellifera*). Gut Pathog..

[20] Audisio M.C., Benítez-Ahrendts M.R. (2011). *Lactobacillus johnsonii* CRL1647, isolated from *Apis mellifera* L. bee-gut, exhibited a beneficial effect on honeybee colonies. Benefic. Microbes.

[21] Corby-Harris V., Maes P., Anderson K.E. (2014). The bacterial communities associated with honey bee (*Apis mellifera*) foragers. PLoS ONE.

[22] Endo A., Salminen S. (2013). Honeybees and beehives are rich sources for fructophilic lactic acid bacteria. Syst. Appl. Microbiol..

[23] Anderson K.E., Sheehan T.H., Mott B.M., Maes P., Snyder L., Schwan M.R., Walton A., Jones B.M., Corby-Harris V. (2013). Microbial ecology of the hive and pollination landscape: Bacterial associates from floral nectar, the alimentary tract and stored food of honey bees (*Apis mellifera*). PLoS ONE.

[24] Huang R.L., Yin Y.L., Wu G.Y., Zhang Y.G., Li T.J., Li L.L., Li M.X., Tang Z.R., Zhang J., Wang B. (2005). Effect of dietary oligochitosan supplementation on ileal digestibility of nutrients and performance in broilers. Poult. Sci..

[25] Pokusaeva K., Fitzgerald G.F., van Sinderen D. (2011). Carbohydrate metabolism in *Bifidobacteria*. Genes Nutr..

[26] Mazzei M., Fronte B., Sagona S., Carrozza M.L., Forzan M., Pizzurro F., Bibbiani C., Miragliotta V., Abramo F., Millanta F. (2016). Effect of 1,3-1,6 β-Glucan on Natural and Experimental Deformed Wing Virus Infection in Newly Emerged Honeybees (*Apis mellifera ligustica*). PLoS ONE.

[27] Vetvicka V., Fernandez-Botran R. (2018). β-Glucan and parasites. Helminthologia.

[28] Saltykova E.S., Gaifullina L.R., Kaskinova M.D., Gataullin A.R., Matniyazov R.T., Poskryakov A.V., Nikolenko A.G. (2018). Effect of chitosan on development of *Nosema apis* Microsporidia in honey bees. Microbiology.

[29] Buddington K.K., Donahoo J.B., Buddington R.K. (2002). Dietary oligofructose and inulin protect mice from enteric and systemic pathogens and tumor inducers. J. Nutr..

[30] Hansen C.H.F., Frøkiær H., Christensen A.G., Bergström A., Licht T.R., Hansen A.K., Metzdorff S.B. (2013). Dietary xylooligosaccharide downregulates IFN-γ and the low-grade inflammatory cytokine IL-1β systemically in mice. J. Nutr..

[31] Younes H., Garleb K., Behr S., Rémésy C., Demigné C. (1995). Fermentable fibres or oligosaccharides reduce urinary nitrogen excretion by increasing urea disposal in the rat cecum. J. Nutr..

[32] Ali B.H., Al-Husseni I., Beegam S., Al-Shukaili A., Nemmar A., Schierling S., Queisser N., Schupp N. (2013). Effect of gum arabic on oxidative stress and inflammation in adenine-induced chronic renal failure in rats. PLoS ONE.

[33] Hamdi C., Balloi A., Essanaa J., Crotti E., Gonella E., Raddadi N., Ricci I., Boudabous A., Borin S., Manino A. (2011). Gut microbiome dysbiosis and honeybee health. J. Appl. Entomol..

[34] Sabaté D.C., Cruz M.S., Benítez-Ahrendts M.R., Audisio M.C. (2012). Beneficial effects of *Bacillus subtilis* subsp. *subtilis* Mori2, a honey-associated strain, on honeybee colony performance. Probiot. Antimicrob. Proteins.

[35] Arredondo D., Castelli L., Porrini M.P., Garrido P.M., Eguaras M.J., Zunino P., Antúnez K. (2018). *Lactobacillus kunkeei* strains decreased the infection by honey bee pathogens *Paenibacillus larvae* and *Nosema ceranae*. Benefic. Microbes.

[36] Corby-Harris V., Snyder L., Meador C.A.D., Naldo R., Mott B., Anderson K.E. (2016). *Parasaccharibacter apium*, gen. nov., sp. nov., improves honey bee (Hymenoptera: Apidae) resistance to *Nosema*. J. Econ. Entomol..

[37] El Khoury S., Rousseau A., Lecoeur A., Cheaib B., Bouslama S., Mercier P.-L., Demey V., Castex M., Giovenazzo P., Derome N. (2018). Deleterious interaction between honeybees (*Apis mellifera*) and its microsporidian intracellular parasite *Nosema ceranae* was mitigated by administrating either endogenous or allochthonous gut microbiota strains. Front. Ecol. Evol..

[38] Andrearczyk S., Kadhim M.J., Knaga S. (2014). Influence of a probiotic on the mortality, sugar syrup ingestion and infection of honeybees with *Nosema* spp. under laboratory assessment. Med. Weter..

[39] Schmidt K., Engel P. (2016). Probiotic treatment with a gut symbiont leads to parasite susceptibility in honey bees. Trends Parasitol..

[40] Mehr M.A., Shargh M.S., Dastar B., Hassani S., Akbari M.R. (2007). Effect of different levels of protein and Protexin on broiler performance. Int. J. Poult. Sci..

[41] Naseri K.G., Rahimi S., Khaki P. (2012). Comparison of the effects of probiotic, organic acid and medicinal plant on *Campylobacter jejuni* challenged broiler chickens. J. Agric. Sci. Technol..

[42] Gary N.E., Marston J.M. (1976). Vacuum apparatus for collecting honey bees Hymenoptera-Apidae and other insects in trees. Ann. Entomol. Soc. Am..

[43] Cantwell G.E. (1970). Standard methods for counting *Nosema* spores. Am. Bee J..

[44] Hamiduzzaman M.M., Guzman-Novoa E., Goodwin P.H. (2010). A multiplex PCR assay to diagnose and quantify *Nosema* infections in honey bees (*Apis mellifera*). J. Invertebr. Pathol..

[45] Maistrello L., Lodesani M., Costa C., Leonardi F., Marani G., Caldon M., Mutinelli F., Granato A. (2008). Screening of natural compounds for the control of *Nosema* disease in honeybees (*Apis mellifera*). Apidologie.

[46] McGowan J., De la Mora A., Goodwin P.H., Habash M., Hamiduzzaman M.M., Kelly P.G., Guzman-Novoa E. (2016). Viability and infectivity of fresh and cryopreserved *Nosema ceranae* spores. J. Microbiol. Methods.

[47] Borges D., Guzman-Novoa E., Goodwin P.H. (2020). Control of the microsporidian parasite *Nosema ceranae* in honey bees (*Apis mellifera*) using nutraceutical and immuno-stimulatory compounds. PLoS ONE.

[48] Klassen S. (2018). Effects of Prebiotics and Probiotics on the Parasitic Microsporidium *Nosema ceranae* and Honey Bee (*Apis mellifera*) Health at the Individual and Colony Levels. Ph.D. Thesis.

[49] Ballal A., Bobbala D., Qadri S.M., Föller M., Kempe D., Nasir O., Saeed A., Lang F. (2011). Anti-malarial effect of gum arabic. Malar. J..

[50] Zentek J., Gärtner S., Tedin L., Männer K., Mader A., Vahjen W. (2013). Fenugreek seed affects intestinal microbiota and immunological variables in piglets after weaning. Br. J. Nutr..

[51] Maggi M., Negri P., Plischuk S., Szawarski N., De Piano F., De Feudis L., Eguaras M., Audisio C. (2013). Effects of the organic acids produced by a lactic acid bacterium in *Apis mellifera* colony development, *Nosema ceranae* control and fumagillin efficiency. Vet. Microbiol..

[52] Porrini M.P., Audisio M.C., Sabaté D.C., Ibarguren C., Medici S.K., Sarlo E.G., Garrido P.M., Eguaras M.J. (2010). Effect of bacterial metabolites on microsporidian *Nosema ceranae* and on its host *Apis mellifera*. Parasitol. Res..

[53] Tajabadi N., Mardan M., Saari N., Mustafa S., Bahreini R., Manap M.W.A. (2013). Identification of *Lactobacillus plantarum*, *Lactobacillus pentosus* and *Lactobacillus fermentum* from honey stomach of honeybee. Braz. J. Microbiol..

[54] Pătruică S., Huţu I. (2013). Economic benefits of using prebiotic and probiotic products as supplements in stimulation feeds administered to bee colonies. Turk. J. Vet. Anim. Sci..

[55] Pătruică S., Mot D. (2012). The effect of using prebiotic and probiotic products on intestinal micro-flora of the honeybee (*Apis mellifera carpatica*). Bull. Entomol. Res..

[56] Evans J.D., Lopez D.L. (2004). Bacterial probiotics induce an immune response in the honey bee (Hymenoptera: Apidae). J. Econ. Entomol..

[57] Yoshiyama M., Wu M., Sugimura Y., Takaya N., Kimoto-Nira H., Suzuki C. (2013). Inhibition of *Paenibacillus larvae* by lactic acid bacteria isolated from fermented materials. J. Invertebr. Pathol..

[58] Bowen J.M., Stringer A.M., Gibson R.J., Yeoh A.S.J., Hannam S., Keefe D.M.K. (2007). VSL#3 probiotic treatment reduces chemotherapy-induced diarrhea and weight loss. Cancer Biol. Ther..

[59] Belhadj H., Harzallah D., Khennouf S., Dahamna S., Bouharati S., Baghiani A. (2010). Isolation, identification and antimicrobial activity of lactic acid bacteria from Algerian honeybee collected pollen. Acta Hortic..

[60] Aufauvre J., Misme-Aucouturier B., Viguès B., Texier C., Delbac F., Blot N. (2014). Transcriptome analyses of the honeybee response to *Nosema ceranae* and insecticides. PLoS ONE.

[61] Rubanov A., Russell K.A., Rothman J.A., Nieh J.C., McFrederick Q.S. (2019). Intensity of *Nosema ceranae* infection is associated with specific honey bee gut bacteria and weakly associated with gut microbiome structure. Sci. Rep..

